# Selection of circulating PD-1+ lymphocytes from cancer patients enriches for tumor-reactive and mutation-specific lymphocytes

**DOI:** 10.1186/2051-1426-3-S2-O2

**Published:** 2015-11-04

**Authors:** Alena Gros, Eric Tran, Maria R Parkhurst, Pasetto Anna, Sadia Ilyas, Todd D Prickett, Jared J Gartner, Paul F Robbins, Jessica S Crystal, Kasia Trebska-Mcgowan, John R Wudnerlich, James C Yang, Steven A Rosenberg

**Affiliations:** 1 National Cancer Institute, National Institutes of Health, Bethesda, MD, USA; 2 Surgery Branch, National Cancer Institute, National Institutes of Health, Bethesda, MD, USA; 3 National Institutes of Health, Bethesda, MD, USA

## Background

T cells targeting unique somatic mutations appear to play an important role in the antitumor responses observed following T cell transfer, and isolation of mutation-specific lymphocytes and T-cell receptors has become a major obstacle to the development of more effective immunotherapies. The detection of tumor-reactive and mutation-specific cells in patients with cancer has been largely restricted to tumor-infiltrating lymphocytes, but mutation-specific cells are thought to be far less prevalent in peripheral blood, a more accessible and abundant source of T cells. We recently reported that expression of PD-1 identifies the patient-specific repertoire of tumor-reactive cells infiltrating melanoma tumors. Given these findings, we explored the utility of PD-1 expression on peripheral blood lymphocytes to detect and enrich for tumor and neoantigen specific lymphocytes.

## Methods

To this end, peripheral blood CD8+ lymphocytes were separated based on the expression of PD-1 into CD8+PD-1- and CD8+PD-1+ and CD8+PD-1hi cells, and expanded in vitro for 15 days. Circulating T cell subsets were subsequently screened for recognition of mutated antigens identified by whole exome sequencing using a high throughput and personalized approach that enables the expression of all the potential tumor neoantigens in the autologous antigen-presenting cells. In addition, recognition of shared melanoma differentiation antigens and cancer germline antigens was also evaluated.

## Results

PD-1+ lymphocytes represented a small percentage of all the circulating CD8+ cells in patients with metastatic melanoma. We found that selection of CD8+PD-1+ lymphocytes circulating in peripheral blood, but not the CD8+ or CD8+PD-1- cells, led to direct enrichment of tumor-reactive cells from peripheral blood in all four patients studied. In three out of four melanoma patients, the peripheral blood CD8+PD-1+ and PD-1hi cells contained mutation-specific lymphocytes targeting 3, 3 and 1 unique patient-specific neoantigens, respectively. In addition, circulating CD8+PD- 1+ and PD-1hi lymphocytes from all four patients evaluated were also enriched in T cells targeting at least one cancer germline antigen, including NY-ESO-I, MAGE-A3, and SSX2. Neither mutation-specific nor cancer germline-specific lymphocytes were detected in the peripheral blood CD8+ or the CD8+PD-1- populations.

## Conclusion

Our findings provide evidence that peripheral blood CD8+PD-1+ from cancer patients are enriched in naturally-occurring tumor-reactive and mutation-specific cells and provide a novel strategy to develop personalized T cell based therapies to treat cancer.

